# A comparison of pharmacometric software programs for atezolizumab population pharmacokinetic simulation

**DOI:** 10.1007/s00228-025-03974-9

**Published:** 2026-01-19

**Authors:** Yi Zeng, Oluwatobi Arisa, Natalia Corvalan, Francis Bateman, Keith Schmidt, Cody Peer, William D. Figg

**Affiliations:** 1 Clinical Pharmacology Laboratory, Clinical Center, NIH, Bethesda, MD USA; 2 Clinical Pharmacology Program, National Cancer Institute, NIH, Bethesda, MD USA; 3 Clinical Pharmacology Program, National Cancer Institute, 10 Center Dr, Room 5A03, Bethesda, MD 20892 USA

**Keywords:** Population pharmacokinetics, Oncology, Clinical trials, Modeling & simulation, Immunopharmacology

## Abstract

**Purpose:**

We compare our experience with three pharmacometric modeling workflows for simulating alternative dosing regimens of atezolizumab: (1) the gold-standard, NONMEM software used in combination with R, (2) the R-based package RxODE, and (3) the recently developed Julia-based software Pumas, discussing the advantages and limitations of each.

**Methods:**

Our prior work demonstrated that an extended-interval dosing regimen (840 mg q6w) following two standard loading doses maintained efficacy while having a nonsignificant exposure-response relationship with adverse events. In the original analysis, the virtual population was generated in R, simulations performed using NONMEM, and data analysis and visualization then conducted in R. In the present study, we perform the full workflow within R using RxODE for simulation and also recreate this workflow using Pumas in Julia. Pharmacokinetic parameters and graphical output, as well as the processing speed for each method were compared.

**Results:**

All three approaches generated comparable virtual populations, key exposure metrics of CMAX, CMIN, and Weekly AUC, and data visualizations of the simulated serum concentrations. However, there were differences in how quickly each software simulated the entire seven cycle dataset, with Pumas simulating 33,273 obs/second, NONMEM 4,782 obs/sec, and RxODE 251 obs/sec. Due to this large difference, the dataset was broken into individual cycles, where NONMEM and RxODE performed comparably at 2041-3337 obs/sec, while Pumas simulated 48,122-69,168 obs/sec.

**Conclusion:**

All three software produced comparable results. Ultimately, the choice should be based on the modeler’s specific needs and limitations.

**Supplementary Information:**

The online version contains supplementary material available at 10.1007/s00228-025-03974-9.

## Introduction

Atezolizumab, a humanized monoclonal antibody targeting PD-L1, is an immune checkpoint inhibitor approved as a first-line treatment for metastatic non-small cell lung cancer (mNSCLC) exhibiting high PD-L1 expression [1, 2]. It is also approved for locally advanced or metastatic urothelial cancer (mUC) [3], metastatic melanoma [4], hepatocellular carcinoma [5], and other indications [6, 7], reflecting its versatility and effectiveness as a therapeutic agent. The currently recommended doses for atezolizumab are 840 mg every 2 weeks (q2w), 1200 mg every 3 weeks (q3w), or 1680 mg every 4 weeks (q4w) [7]. However, at 1200 mg q3w, a significant (N = 1007, *p* = 0.027) trend of increased incidence of adverse events of special interest (AESI) was observed with increasing atezolizumab exposure [8]. In our prior study, we replicated the logistic regression model with simulated exposures (steady state AUCs from 1200 mg q3w) vs. AESI and observed a similar trend (*p* = 0.022; α = 0.05) [6].

Consequently, in our previous study, an alternative lower dosage regimen was proposed to maintain concentrations above 6 µg/mL, the minimum effective concentration (MEC) of atezolizumab in combination with bevacizumab, as defined by the sponsor [6]. This recommendation was based on population pharmacokinetic simulations from an established model and a virtual patient population [8]. Simulations were originally conducted using the industry standard, NONMEM, which was coupled with the R software package PKNCA for pharmacokinetic (PK) analysis and ggplot2 for data visualization. The combined use of NONMEM and R necessitates proficiency in both platforms and imposes additional time constraints due to the laborious analysis of large datasets. Although R can be employed independently for simulation and visualization, this approach is also very time consuming. To demonstrate this, the R package RxODE was used in this study for comparison, however, it is important to note that multiple R packages exist for simulation.

A novel software, Pumas [9], was developed in Julia to streamline PK analysis and was employed in this study to simulate alternative dosage regimens for atezolizumab. Herein, we compare the application of Pumas with NONMEM and R in conducting a comprehensive PK analysis using a previously published dataset, evaluating the capabilities of Pumas. Our comparison includes the assessment of processing times when employing NONMEM and R in tandem for simulation and visualization, respectively, as well as the use of R or Pumas for both processes.

## Methods

In our prior paper, alternative extended-interval regimens were simulated using a previously published population PK model in the FDA CDER clinical pharmacology review for atezolizumab in NSCLC [6, 8]. A virtual patient population was generated using R 4.3.3 (www.r-project.org), and Monte Carlo simulations were performed using NONMEM 7.5.1 (64-bit, ICON Development Solutions, Ellicott City, MD) and Pirana 2.9.9 (Certara, Princeton, NJ) with PsN and Xpose. Graphs and statistics were then generated in R using ggplot2, tidyverse, and PKNCA packages.

In this study, a recreation of the prior established population PK model for all dosing regimens was performed utilizing Pumas 2.5.1 (PumasAI, Dover, Delaware), a pharmacometric software platform created in Julia 1.75.2 (https://julialang.org/) [6, 8]. The same proposed dosing regimens, dense simulations, graphics, and statistical output were performed using Pumas and the corresponding data visualization package in Julia using AlgebraOfGraphics. Separately, the RxODE package was used to perform the Monte Carlo simulations instead of NONMEM, with graphs and statistics generated using the same R packages mentioned above.

Virtual patient population demographics can be found in Table 1. A seed number was set to ensure consistent results. The virtual patient population (*n* = 1000) demographics were generated by randomly simulating ages from 20 to 80 years old and with even odds for sex. Body weight was dependent on age and sex (Supplemental Material).

$$\:{Weight}_{Female}=65+0.75\times\:\left(Age-40\right)+\:N(0,\:3.5)$$

$$\:{Weight}_{Male}=85+0.75\times\:\left(Age-40\right)+N(0,\:10)\:$$

**Table 1:** Virtual patient population demographics

Characteristic	NONMEM (*n* = 1000)	Pumas (*n* = 1000)	RxODE (*n* = 1000)
Sex, n (%)			
Male	469 (46.9%)	488 (48.8%)	487 (48.7%)
Female	531 (53.1%)	512 (51.2%)	513 (51.3%)
Age (years)^†^	44.6 [20.1–79.8]	49.0 [20–80]	51 [20–80]
Body Weight (kg)^†^	79.6 [44.3–136]	82.9 [43.2–133.9.2.9]	83.2 [43.2–130]
Baseline albumin (g/L)^†^	42.2 [32.3–53.7]	42.1 [28.1–54.2]	42.0 [28.4–53.4]
Baseline tumor size (mm)^†^	68.9 [9.54–686]	69.3 [5.6–536.1.6.1]	67.5 [7.67–625]
Anti-Drug Antibodies, n (%)			
No	567 (56.7%)	591 (59.1%)	617 (61.7%)
Yes	433 (43.3%)	409 (40.9%)	383 (38.3%)

^†^median [min-max]

There was also a 40% probability that the virtual patient would form anti-drug antibodies. Albumin and baseline tumor burden were simulated using a log-normal distribution with a mean of 42 g/dL and 4.2 mm^3^, respectively, based on the baseline median found in Stroh et al. [10]

The standard dosing regimen of 1200 mg Q3W was simulated in addition to three alternative dosing regimens that maintained the MEC of 6 µg/mL selected in our prior paper in the 1000-virtual-patient data set [6]. Extended interval dosing regimens were: 2 cycles of loading doses at either 840 mg Q2W, 1200 mg Q3W, or 1680 mg Q6W followed by extended interval dosing of 840 mg Q6W for a total of 7 cycles to achieve steady state. Summary statistics of the C_MAX_, C_MIN_, and the weekly AUC of the first cycle and final cycle at steady state were calculated, including the average, median, standard deviation, and the 5th, 50th, and 95th percentiles of the concentrations at each time point from each of the 1000 virtual patients. The graphics provided highlight the median with 90% PI for the time after the first and seventh cycles.

All simulations were conducted on the same computer system to compare the three modeling packages—Pumas, NONMEM, and RxODE—and ensure fairness and accuracy. This was conducted on an Intel i7-1270P CPU (16 threads), 32.0 GB of RAM, and Windows 11 Enterprise. We evaluated processing speed by comparing the number of observations each software simulated over run time.

## Results

**Fig. 1:**
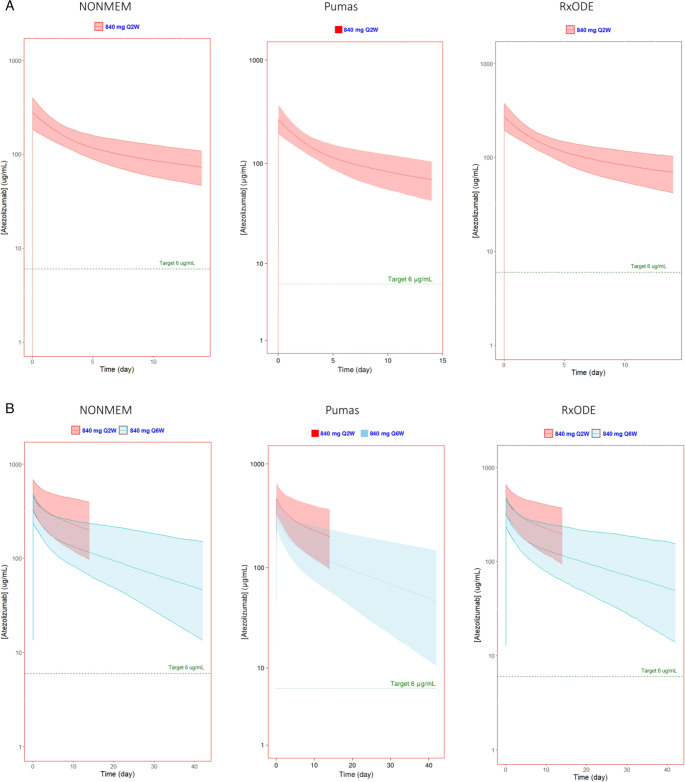
Simulated atezolizumab serum concentrations of standard 840 mg q2w followed by 840 mg q6w generated using NONMEM, Pumas, and RxODE visualized using ggplot2 in R (NONMEM and RxODE) or AlgebraOfGraphics in Julia (Pumas). Predicted (**A**) first dose and (**B**) seventh dose (at steady state) showed extended interval dosing maintains a serum concentration well above the target concentration comparable to that of the current recommended doses. The solid line represents the median and the shaded region represents the 90% prediction interval

**Fig. 2:**
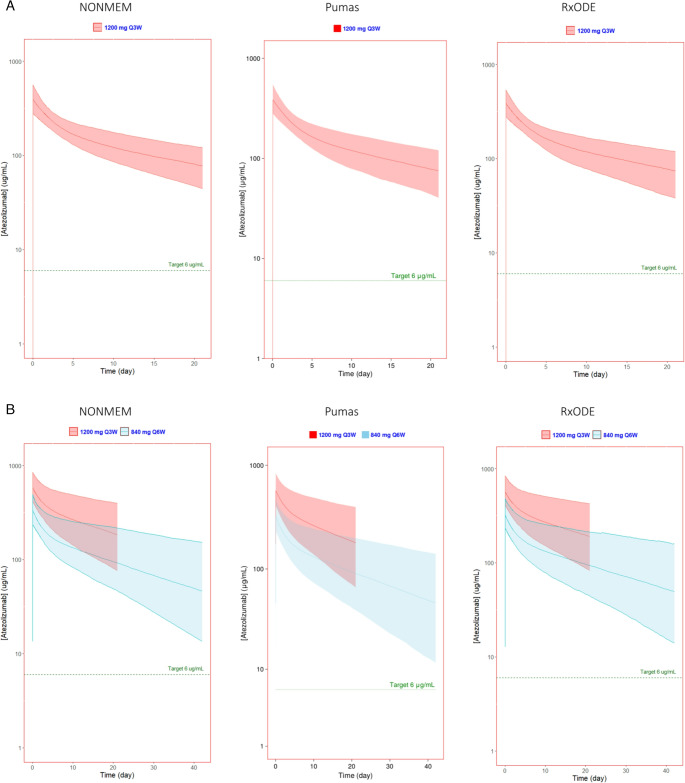
Simulated atezolizumab serum concentrations of standard 1200 mg q3w followed by 840 mg q6w generated using NONMEM, Pumas, and RxODE visualized using ggplot2 in R (NONMEM and RxODE) or AlgebraOfGraphics in Julia (Pumas). Predicted (**A**) first dose and (**B**) seventh dose (at steady state) showed extended interval dosing maintains a serum concentration well above the target concentration comparable to that of the current recommended doses. The solid line represents the median and the shaded region represents the 90% prediction interval

**Fig. 3:**
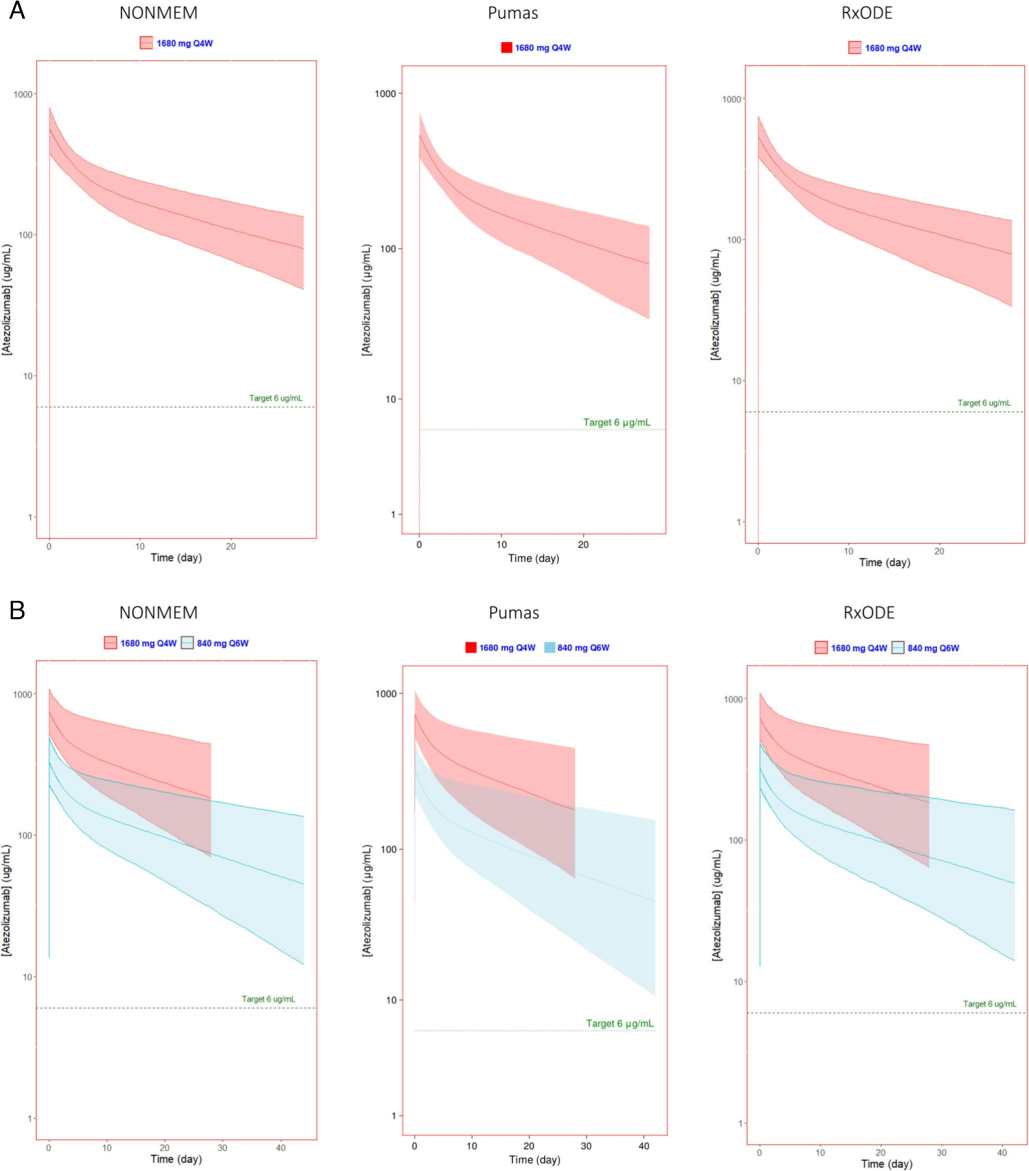
Simulated atezolizumab serum concentrations of standard 1680 mg q4w followed by 840 mg q6w generated using NONMEM, Pumas, and RxODE visualized using ggplot2 in R (NONMEM and RxODE) or AlgebraOfGraphics in Julia (Pumas). Predicted (**A**) first dose and (**B**) seventh dose (at steady state) showed extended interval dosing maintains a serum concentration well above the target concentration comparable to that of the current recommended doses. The solid line represents the median and the shaded region represents the 90% prediction interval

The pharmacokinetic parameters calculated by all three simulation methods for the standard dosing regimen and three extended-interval regimens showed similar results as expected (Table 2, Supplemental Material). Additionally, the simulated cycle 1 and 7 serum atezolizumab concentration profiles for each software platform are presented in Figs. 1, 2 and 3. Figure 1 shows the simulated concentrations vs. time of 840 mg Q2W x2 followed by 840 mg Q6W, Fig. 2 shows 1200 mg Q3W x2 followed by 840 mg Q6W, and Fig. 3 shows 1680 mg Q4W followed by 840 mg Q6W.

**Table 2:** C_max_, C_min_ and weekly AUC values per cycle from Pumas, NONMEM and RxODE

	Pumas			NONMEM			RxODE		
Cycle	C_MAX_ (µg/mL)	C_MIN_ (µg/mL)	Weekly AUC (µg*day/mL)	C_MAX_ (µg/mL)	C_MIN_ (µg/mL)	Weekly AUC (µg*day/mL)	C_MAX_ (µg/mL)	C_MIN_ (µg/mL)	Weekly AUC (µg*day/mL)
1200 mg q3w									
1	389 (280–548)	73.1 (40.4–120)	980 (722–1342)	394 (278–559)	75.6 (44.5–121)	1003 (755–1342)	386 (279–536)	71.6 (38.1–119)	967 (719–1288)
7	572 (406–831)	177 (66.1–397)	1985 (1086–3502)	583 (414–847)	180 (75.9–396)	2014 (1169–3535)	577 (409–843)	187 (82.7–425)	2043 (1215–3675)
840 mg q2w x2, 840 mg q6w x5									
1	269 (193–371)	68.5 (43.4–105)	811 (628–1073)	275 (184–397)	71.2 (46.4–108)	834 (620–1131)	270 (196–375)	67.7 (41.9–103)	808 (621–1047)
7	328 (234–473)	45.7 (12.3–139)	760 (417–1435)	336 (235–487)	46.9 (13.5–150)	774 (441–1562)	328 (233–484)	47.5 (14.0–156.0)	771 (425–1559)
1200 mg q3w x2, 840 mg q6w x5									
1	389 (280–548)	73.1 (40.4–120)	980 (722–1342)	394 (278–559)	75.6 (44.5–121)	1003 (755–1342)	386 (279–536)	71.6 (38.1–119)	967 (719–1288)
7	328 (234–473)	45.8 (12.3–140)	763 (417–1448)	327 (217–492)	46.6 (13.4–157)	763 (411–1594)	329 (234–484)	47.7 (14.0–159.0)	774 (425–1609)
1680 mg q4w x2, 840 mg q6w x5									
1	536 (381–755)	75.8 (34.0–137.0)	1179 (818–1632)	552 (375–793)	78.1 (40.7–135)	1203 (869–1651)	540 (391–751)	74.9 (33.4–136)	1171 (846–1615)
7	329 (234–474)	46.0 (12.3–142)	766 (418–1462)	332 (227–491)	46.5 (13.6–140)	771 (436–1464)	330 (235–486)	48.0 (14.0–162.0)	745 (425–1625)

All values are geometric means (90% PI) from 1000 virtual patients

The amount of time to simulate all seven cycles at once was significantly greater than simulating each cycle individually (Table 3). While simulating all seven cycles of 1,000 virtual patients, Pumas took about 51 s to simulate 1,688,000 datapoints (33,273 obs/sec), NONMEM took about 5.9 min (4,782 obs/sec), and RxODE took about 112 min (251 obs/sec). Due to this difference in very large datasets, the comparison for each method broke the total simulation down to individual cycles, focusing on cycles 1 and 7. Cycle 1 of the first extended interval regimen (840 mg Q2W x2, 840 mg Q6W x5) took Pumas less than a second to simulate 344,000 observations and another 5 s to convert and save the simulation results into a usable data frame (54,442 obs/sec). Comparatively, it took NONMEM a total of 47 s to simulate 145,000 observations (3,104 obs/sec) and RxODE a total of 44 s to simulate 146,000 observations (3,337 obs/sec).

**Table 3:** A comparison of processing speed between Pumas, NONMEM, and RxODE in terms of number of observations simulated per second

Processing speed (Obs/sec)			
Regimen	Pumas	NONMEM	RxODE
840 mg q2w X2, 840 mg q6w Cycle 1	54,442	3104	3337
840 mg q2w X2, 840 mg q6w Cycle 7	55,321	3101	2041
1200 mg q4w X2, 840 mg q6w Cycle 1	69,168	3137	2942
1200 mg q4w X2, 840 mg q6w Cycle 7	61,807	2858	2060
1680 mg q4w X2, 840 mg q6w Cycle 1	48,122	2937	2597
1680 mg q4w X2, 840 mg q6w Cycle 7	57,637	2881	2068

## Discussion

Simulations using NONMEM, Pumas, and RxODE yielded comparable exposure metrics in all four dosing regimens. However, there is a significant difference in processing speed between newer software like Pumas and older software like NONMEM. It is important to note that the duration of each simulation may vary between runs, and slight delays can occur when executing multiple lines of code consecutively. However, despite this, Pumas was at least ten times faster than either NONMEM or RxODE. This is likely due to backend optimization in the ODE solver, including a better compiler for ODE function and size, and utilizing options in the Julia package differentialEquations for efficient integration methods and better stability.^9^ Comparatively, while RxODE performed equivalently with NONMEM on smaller datasets, it was the slowest at simulating the full dataset. Although RxODE better optimizes ODE models in R compared to general-purpose differential equation solvers by automatically translating them into C, certain efficiency steps are not automatically built in [11]. The user must take additional steps to further speed up simulations, with examples available in the package resources, and should have some prior knowledge of performance considerations to do so. While this study does not evaluate or compare the technical aspects responsible for the marked difference in computational speed, it will be important to continue to explore how pharmacometric software platforms continue to optimize workflows.

In addition, Pumas also showed the ability to handle and simulate large data sets efficiently. Coupled with its AI capability, Pumas could handle large and diverse data sets that would otherwise take far longer and manual human intervention to data wrangle. However, NONMEM is more established, has more supporting software, and guidance for pharmacokinetic analysis, such as Pirana with PsN and Xpose that provide a more user-friendly graphical user interface and provides a bridge between results generated by NONMEM and statistical and visual output using R code. On the other hand, a major benefit of using RxODE is that it is an open-source program, while both NONMEM and Pumas require paid licenses. Using RxODE would allow for those with budgetary constraints to conduct similar pharmacometric simulations with industry standard alternatives, although it may lack some of the validation aspects provided by commercially licensed software. Additionally, there are multiple R packages that can also be used for population PK simulations in R, such as mrgsolve, PKPDsim, and ubiquity, with many more serving as bridges between other software and R.

Another benefit of both Pumas and R, is that the entire process, from simulation, NCA analysis, data wrangling, and graphical analysis, can be done in the same program. This simplifies the entire process by requiring familiarity with only one coding language and software, as well as requiring only one license or, in the case of R, no license. NONMEM, however, requires rigid datasets and data wrangling and visualization in a program like R, as it only covers the simulation aspect.

In conclusion, while there are advantages and limitations with each software, the final choice of modeling software depends on the user’s specific requirements and constraints.

## Supplementary Information

Below is the link to the electronic supplementary material.

Supplementary Material 1

## Data Availability

The simulated data can be shared by sending requests to [FiggW@nih.gov](mailto: FiggW@nih.gov).
