# Supplementary material for: A comparison of pharmacometric software programs for atezolizumab population pharmacokinetic simulation
Source: Eur J Clin Pharmacol. 2026 Jan 19;82(2):42. doi: 10.1007/s00228-025-03974-9 (PMC12815976; doi:10.1007/s00228-025-03974-9)
Supplement: Supplementary file 1 — Supplementary Material 1 [file 228_2025_3974_MOESM1_ESM.docx]

**Atezolizumab Virtual Patient (n=1000) Generation for NONMEM and RxODE**

###### Simulate population

set.seed(12345)

n <- 1000

Age <- round(runif(nsub, min=20, max=80)) #years

SEX <- round(runif(nsub, min=0, max=1)) #0=male, 1=female

BWf <- 65 + 0.75 * (Age - 40) + rnorm(nsub, 0, sd=3.5)

BWm <- 85 + 0.75 * (Age - 40) + rnorm(nsub, 0, sd=10)

BW <- ifelse(SEX == 1, BWf, BWm) #Weight (kg) based on age and sex

ATAG <-rbinom(nsub, 1, 0.4) #0 = absence of antitherapy antibodies,

# 1 = presence of ATAG

Alb <-rnorm(nsub, 42, sd=3.5) #Albumin (g/dL)

BTS <-rlnorm(nsub, 4.2, sd=0.7) #Baseline Tumor Burden (mm)

Covs <- tibble(id = seq_along(Age), Age = Age, SEX = SEX,

BW = BW, ATAG = ATAG, Alb = Alb, BTS = BTS)

**Atezolizumab Virtual Patient (n=1000) Generation for Pumas**

###### Simulate population

Random.seed!(12345)

rng = Random.Xoshiro(12345)

function choose_mltpl_covariates(rng)

Age = round(rand(rng, 20:80))

SEX = rand(rng, Bernoulli(0.5))

BW = ifelse.(SEX .== 1,

65 .+ 0.75 .* (Age .- 40) .+ rand.(rng, Normal(0, 3.5)),

85 .+ 0.75 .* (Age .- 40) .+ rand.(rng, Normal(0, 10)))

ATAG = rand(rng, Bernoulli(0.4))

Alb = rand(rng, Normal(42, 3.5))

BTS = rand(rng, LogNormal(4.2, 0.7))

return (; Age, SEX = Int.(SEX), BW, ATAG = Int.(ATAG), Alb,

BTS)

end

**Atezolizumab Simulation Model: Duplicated from FDA CDER Clinical Pharmacology and Biopharmaceutics Review; 761041Orig1s000**

**NONMEM**

;; 1. Based on: run4

;; 2. Description: 840mg q4w - C7

;; x1. Author: Cody_Peer

; Simulation of 840 mg atezolizumab q4wk in 1000 virtual patients

$PROBLEM 840mg q4w - C7

$INPUT ID TIME AMT CMT RATE DV MDV EVID ISF WT AGE ATAG ALB BTS

$DATA SimCov_Atezo_n1000_NM_840mgQ4W_C7.csv ; *changes with each scenario*

IGNORE=@

$SUBROUTINES ADVAN3 TRANS4

$PK

TVCL = THETA(3) *(WT/77)**THETA(7) *(ALB/40)**THETA(8) *(BTS/63)**THETA(9) * (THETA(10)**ATAG) * EXP(ETA(1))

TVV1 = THETA(4) *(WT/77)**THETA(11) * (ALB/40)**THETA(12) * (THETA(13)**ISF)

TVQ = THETA(5)

TVV2 = THETA(6) *(THETA(14)**ISF)

IMAX = THETA(15) * EXP(ETA(4))

T50 = THETA(16)

GMA = THETA(17)

CL = TVCL * EXP((IMAX * (TIME**GMA))/(T50**GMA + TIME**GMA))

V1 = TVV1 * EXP(ETA(2))

Q = TVQ

V2 = TVV2 * EXP(ETA(3))

S1 = V1

$THETA

(0.034) FIX ; THETA(1), exponential error

(18.1) FIX ; THETA(2), additive error

(0.23) FIX ; THETA(3), CL

(3.25) FIX ; THETA(4), V1

(0.603) FIX ; THETA(5), Q

(2.88) FIX ; THETA(6), V2

(0.668) FIX ; THETA(7), [CL~BW]

(-0.901) FIX ; THETA(8), [CL~ALB]

(0.116) FIX ; THETA(9),[CL~BTS]

(1.158) FIX ; THETA(10),[CL~ATAG]

(0.533) FIX ; THETA(11),[V1~WT]

(-0.345) FIX ; THETA(12),[V1~ALB]

(0.896) FIX ; THETA(13),[V1~SEX (ISF)]

(0.707) FIX ; THETA(14),[V2~SEX (ISF)]

(-0.193) FIX ; THETA(15),IMAX

(62.8) FIX ; THETA(16),T50

(2.67) FIX ; THETA(17),GAMMA

$OMEGA

0.069169 FIX ; ETA(1), IIV CL

0.029584 FIX ; ETA(2), IIV V1

0.123904 FIX ; ETA(3), IIV V2

0.804609 FIX ; ETA(4), IIV IMAX

$SIGMA

1 FIX

$ERROR

IPRED = F

IRES = DV-IPRED

W = SQRT(THETA(1)**2*IPRED**2 + THETA(2)**2)

IF (W.EQ.0) W = 1

IWRES = IRES/W

Y = IPRED+W*ERR(1)

$SIM (20100922 NEW) (20100922 UNIFORM) ONLYSIM SUBPROBLEMS=1

$TABLE ID TIME MDV EVID DV IPRED TVCL CL FILE=sdtab006

;----------------------------------

**RxODE**

##### Model Object

mod <- rxode2({

Vc = tvvc * (BW/77)^tvBWVc * (Alb/40)^tvAlbVc * (tvSexVc)^SEX * exp(eta.Vc);

Vp = tvvp * (tvSexVp)^SEX * exp(eta.Vp);

CL = tvcl * (BW/77)^tvBWCL * (Alb/40)^tvAlbCL * (BTS/63)^tvBTSCL * (tvATAGCL)^ATAG * exp(eta.CL);

Q = tvq;

Imax = tvImax * exp(eta.IMAX);

CLt = CL * exp((Imax * time^tvGamma)/(tvT50^tvGamma + time^tvGamma));

C1 = centr/Vc;

C2 = peri/Vp;

d/dt(centr) = - Q*C1 + Q*C2 - CLt*C1;

d/dt(peri) = Q*C1 - Q*C2;

cp = C1 + sqrt((C1^2)*(prop.err^2) + (add.err^2));

})

##### Fixed parameters

theta <- c( tvvc = 3.25,

tvvp = 2.88,

tvcl = 0.23,

tvq = 0.603,

tvT50 = 62.8,

tvImax = -0.193,

tvGamma = 2.67,

tvBWCL = 0.668,

tvAlbCL = -0.901,

tvBTSCL = 0.116,

tvATAGCL= 1.158,

tvBWVc = 0.533,

tvAlbVc = -0.345,

tvSexVc = 0.896,

tvSexVp = 0.707)

##### Variability

omega <- lotri(eta.Vc ~ 0.029584, eta.Vp ~ 0.123904, eta.CL ~ 0.069169,

eta.IMAX ~ 0.804609)

sigma <- lotri(add.err ~ 18.1, prop.err ~ 0.034)

**Pumas**

# Model Object

model_full = @model begin

@metadata begin

desc = "Two Compartment Model"

timeu = u"hr"

end

@param begin

"Volume of Central Compartment (L)"

tvvc ∈ RealDomain(lower=0) ## ∈

"Volume of Peripheral Compartment (L)"

tvvp ∈ RealDomain(lower=0)

"Clearance (L/hr)"

tvcl ∈ RealDomain(lower=0)

"Intercompartmental Clearance (L/hr)"

tvq ∈ RealDomain(lower=0)

"Time to 50% Effect"

tvT50 ∈ RealDomain(lower=0)

"Maximum Effect"

tvImax ∈ RealDomain(lower=-1)

"Gamma"

tvGamma ∈ RealDomain(lower=0)

"COV BW on CL"

tvBWCL ∈ RealDomain(lower=0)

"COV Alb on CL"

tvAlbCL ∈ RealDomain(lower=-1)

"COV Baseline Tumor Size on CL"

tvBTSCL ∈ RealDomain(lower=0)

"COV AntiDrug Antibodies on CL"

tvATAGCL ∈ RealDomain(lower=0)

"COV BW on Vc"

tvBWVc ∈ RealDomain(lower=0)

"COV Alb on Vc"

tvAlbVc ∈ RealDomain(lower=-1)

"COV Sex on Vc"

tvSexVc ∈ RealDomain(lower=-10)

"COV Sex on Vp"

tvSexVp ∈ RealDomain(lower=0)

Ω ∈ PDiagDomain(4)

"Proportional RUV"

σ²_prop ∈ RealDomain(lower=0)

"Additive RUV"

σ_add ∈ RealDomain(lower=0)

end

@random begin

η ~ MvNormal(Ω)

end

@covariates BW Alb BTS SEX ATAG

@pre begin

Vc = tvvc * (BW/77)^tvBWVc * (Alb/40)^tvAlbVc *

(tvSexVc)^SEX * exp(η[1])

Vp = tvvp * (tvSexVp)^SEX * exp(η[2])

      CL = tvcl * (BW/77)^tvBWCL * (Alb/40)^tvAlbCL *

(BTS/63)^tvBTSCL * (tvATAGCL)^ATAG * exp(η[3])

Q = tvq

Imax = tvImax * exp(η[4])

CLt = CL * exp((Imax * t^tvGamma)/

(tvT50^tvGamma + t^tvGamma))

end

@dynamics begin

Central' = -(Q/Vc)*Central +

(Q/Vp)*Peripheral -(CLt/Vc)*Central

Peripheral'= (Q/Vc)*Central -(Q/Vp)*Peripheral

end

@derived begin

cp = @. Central/Vc

"""

Observed Concentration (mg/L)

"""

dv ~ @. Normal(cp, σ_add + sqrt(cp^2*σ²_prop))

end

end

# Fixed parameters

param = (tvvc = 3.24,

tvvp = 2.88,

tvcl = 0.009583,

tvq = 0.025125,

tvT50 = 1507.1999,

tvImax = -0.193,

tvGamma = 2.67,

tvBWCL = 0.668,

tvAlbCL = -0.901,

tvBTSCL = 0.116,

tvATAGCL = 1.158,

tvBWVc = 0.533,

tvAlbVc = -0.345,

tvSexVc = 0.896,

tvSexVp = 0.707,

Ω = Diagonal([0.029584, 0.123904, 0.069169, 0.804609]),

σ²_prop = 0.034,

σ_add = 18.1)

**Supplemental Table 1:** **C_MAX_, C_MIN_, and weekly AUC from Pumas, NONMEM, and RxODE with the same virtual population (n=100) and no random effects**

|  | **Pumas** | **NONMEM** | **RxODE** |
| --- | --- | --- | --- |
| **840 mg Q2W x2 - Cycle 1** | | | |
| C_MAX_ (µg/mL) | 269.5 (234.8-326.4) | 269.1 (234.4-325.9) | 269.5 (234.8-326.4) |
| C_MIN_ (µg/mL) | 72.4 (59.0-89.9) | 72.4 (59.0-89.9) | 72.4 (59.0-89.9) |
| Weekly AUC (µg*day/mL) | 818.8 (712.9-962.6) | 818.8 (712.9-962.5) | 818.8 (712.9-962.5) |
